# Genetic Variations Creating MicroRNA Target Sites in the *FXN* 3′-UTR Affect Frataxin Expression in Friedreich Ataxia

**DOI:** 10.1371/journal.pone.0054791

**Published:** 2013-01-30

**Authors:** Simonetta Bandiera, François Cartault, Anne-Sophie Jannot, Elie Hatem, Muriel Girard, Laila Rifai, Clemence Loiseau, Arnold Munnich, Stanislas Lyonnet, Alexandra Henrion-Caude

**Affiliations:** 1 INSERM U781 Hôpital Necker–Enfants Malades, Université Paris Descartes-Sorbonne Cité, Institut Imagine, Paris, France; 2 INSERM U781 Hôpital Necker–Enfants Malades, Paris, France; 3 Département de Génétique, Centre Hospitalier Régional de La Réunion, Saint-Denis, La Réunion, France; 4 Department of Medical Genetics, National Institute of Health, Rabat, Morocco; University of Iowa Carver College of Medicine, United States of America

## Abstract

Friedreich’s ataxia (FRDA) is a severe neurodegenerative disease caused by GAA repeat expansion within the first intron of the *frataxin* gene. It has been suggested that the repeat is responsible for the disease severity due to impaired transcription thereby reducing expression of the protein. However, genotype-phenotype correlation is imperfect, and the influence of other gene regions of the *frataxin* gene is unknown. We hypothesized that FRDA patients may harbor specific regulatory variants in the 3′-UTR. We sequenced the 3′-UTR region of the *frataxin* gene in a cohort of 57 FRDA individuals and 58 controls. Seven single nucleotide polymorphisms (SNPs) out of 19 were polymorphic in our case-control sample. These SNPs defined several haplotypes with one reaching 89% of homozygosity in patients versus 24% in controls. In another cohort of 47 FRDA Reunionese patients, 94% patients were found to be homozygous for this haplotype. We found that this FRDA 3′-UTR conferred a 1.2-fold decrease in the expression of a reporter gene versus the alternative haplotype configuration. We established that differential targeting by miRNA could account for this functional variability. We specifically demonstrated the involvement of miR-124 (i.e hsa-mir-124-3p) in the down-regulation of FRDA-3′-UTR. Our results suggest for the first time that post-transcriptional regulation of frataxin occurs through the 3′-UTR and involves miRNA targeting. We propose that the involvement of miRNAs in a FRDA-specific regulation of frataxin may provide a rationale to increase residual levels of frataxin through miRNA-inhibitory molecules.

## Introduction

Friedreich’s ataxia (FRDA) is the most frequent hereditary ataxia in Western European descent [1]. This progressive autosomal recessive disorder is primarily characterized by neurodegeneration and cardiomyopathy [1]. The onset of symptoms typically occurs around puberty, but earlier and later onsets also exist [1], [2]. FRDA is caused by a deficit of frataxin, a mitochondrial protein involved in iron metabolism and sensitivity to oxidative stress (reviewed in [3]). The most common disease mutation is an expansion of trinucleotide GAA repeats within the first intron of the *frataxin* (*FXN)* gene [4]. The vast majority of patients are homozygous for the GAA expansion harbouring 70 to 1700 repeats, while unaffected individuals usually present 6 to 36 repeats [4]–[6]. Expanded alleles were reported to derive from a single founder chromosome [5], [7]. Therefore, a major risk haplotype at the *FXN*, constituted by several markers, was defined as strongly associated both to expansion and “premutation” intermediates [7].

*In vitro* and *in vivo* data suggest that the expansion mutation results in partial transcriptional inhibition of the *FXN* gene in FRDA, leading to decreased frataxin (reviewed in [8]). In patients homozygous for the expansion mutation, residual levels of frataxin protein were inversely correlated with the size of the GAA repeat on the smaller allele [9], [10]. However, some of those patients presented higher residual frataxin level than expected from their expansion size, suggesting that beside transcriptional regulation multiple mechanisms may regulate the expression of frataxin. So far, very little is known about the regulation of the stability of frataxin transcript and protein. Beyond the proposed post-translational regulation of frataxin by recombinant human erytrhopoietin [11] and by the ubiquitin-proteasome system [12], the post-transcriptional mechanisms controlling frataxin expression are poorly studied.

Post-transcriptional regulation of genes frequently occurs through a set of diverse elements in messenger RNA (mRNA) 3′ untranslated regions (UTRs). Those motifs are bound by regulatory proteins or RNAs, including miRNAs. These endogenous small RNAs modulate many biological cellular functions [13]. Genetic variations in miRNA target sites that disrupt the formation of the miRNA/target duplex can contribute to numerous human diseases [14]. Importantly, dysregulation of miRNAs in the nervous system was suggested to contribute to the mechanisms of neurodegeneration (reviewed in [15]). In FRDA, involvement of miRNAs has been shown to contribute to the cardiac phenotype through the differential targeting of AGTR1 by miR-155 [16]. More recently, a small non-coding RNA, miR-886-3p, which is no longer considered as a miRNA (miRBase, release 16), was found elevated in peripheral blood of FRDA patients and was shown to influence frataxin transcription [17]. Through studying the *FXN* 3′UTR, we sought whether FRDA patients could harbor any specific post-transcriptional regulation of frataxin related to miRNA.

## Materials and Methods

### Ethics Statement

For each patient written informed consent was obtained according to the French Ethics Committee, and all procedures were approved by the Necker Hospital reviewing board.

### Subjects

Patients of either sex diagnosed with FRDA were determined at the molecular level as being homozygous for GAA expansion. Number of repeats ranged from 330 to 1500. One cohort was pediatric (n = 57) with patients followed by the Necker Children’s Hospital. The other cohort was adult (n = 47) with patients followed by the CHR Félix Guyon, Saint-Denis, La Réunion, France. Control subjects of either sex were patients genetically tested at the Necker Children’s Hospital for diseases non-related to FRDA (n = 58).

### PCR Sequencing and Markers

The sequence of human *FXN* 3′-UTR was retrieved from NCBI (NM_000144 and NM_181425) and Ensembl genome browser (ENST00000377270, ENST00000498653, ENST00000396366 and ENST00000484259). Variations of the *FXN* locus were retrieved from the dbSNP database, build 129 (http://www.ncbi.nlm.nih.gov/SNP). In addition to short genetic variations of the 3′-UTR, rs3829062, also referred to as ITR3 due to location in intron 3 of the *FXN* gene, was also genotyped. This SNP has been previously shown to be associated with FRDA [5]. Genomic DNA was kindly provided by Pr Jean-Paul Bonnefont, Hôpital Necker, Paris, and the Centre de Ressources Biologiques, CHR Félix Guyon, Saint-Denis, La Réunion. For genotyping, the sequences of primers used are as follows: FXN_1F, 5′-CCGCAGAGCTCACTAAAGC-3′; FXN_1R, 5′-ATTCATTTTCCCTCCTGGAA-3′; FXN_2F, 5′-TGTCGAAAGCAACTCACACG-3′; FXN_2R, 5′-GAACTATGTCTAGGACCAGG-3′; FXN_3F, 5′-TGTCCAGGGAGACCTAGTGC-3′; FXN_3R, 5′-AGGTTGCTTGACAGGACCAC-3′; FXN_4F, 5′-ATGGTTGATTCCCAGCATTC-3′; FXN_4R, 5′-CAACCTCCACCTCTGGGTTC-3′; ITR3_F, 5′-AAAATGGAAGCATTTGGTAATCA-3′; ITR3_R, 5′-AGTGAACTAAAATTCTTAGAGGG-3′. PCR amplification was performed using reagents from Roche Diagnostics with an annealing temperature range of 55–62°C. PCR products were sequenced using Big Dye Terminator v3.1 (Life Technologies) according to manufacturer’s instructions.

### Cell Culture, Transfection and Luciferase Reporter Assay

HEK-293 and U2OS cell lines were obtained from ATCC (ATCC, Manassas, VA, USA). Cells were grown in Dulbecco’s modified Eagle medium (DMEM) with 10% fetal bovine serum, 100 U/ml penicillin and 100 mg/ml streptomycin at 37°C at an atmosphere of 5% CO_2_. Dual luciferase reporters were generated by inserting the *FXN* 3′-UTR carrying either the FRDA haplotype (FRDA-3′-UTR) or the alternative haplotype configuration (WT-3′-UTR) between the *Not*I and *Xho*I sites of the psiCHECK2 plasmid (Promega). To assess the functionality of *FXN* 3′-UTR, 150 ng of the so-obtained constructs, i.e. plasmid FRDA-3′-UTR and plasmid WT-3′-UTR, respectively, were transfected in either cell lines using Fugene HD (Roche Diagnostics) according to manufacturer’s instructions. Cells transfected with empty psiCHECK2 vector were used as a control. To assess the regulation of *FXN* by hsa-miR-124, HEK-293 cells were transfected with a Fugene HD (Roche Diagnostics)-complexed mixture of 10 nM miRIDIAN mimic hsa-miR-124 (Dharmacon) and 150 ng of plasmid DNA (either plasmid FRDA-3′-UTR or plasmid WT-3′-UTR). The miRIDIAN mimic negative control 1 was used as a negative control. In all transfection experiments, cells were lysed with Passive Lysis Buffer (Promega) thirty-six hours post-transfection and luciferase levels were analyzed using the Dual Luciferase reporter assay (Promega) on a Centro LB960 Microplate Luminometer (Berthold).

### Computational Prediction of miRNA Targets and miRNA Expression

miRNA sequences were retrieved from miRBase registry, release 13. miRNA targeting analysis on the *FXN* 3′-UTR region from both FRDA patient and control were performed using miRDB (http://mirdb.org/miRDB/) and our in-house developed tool MiRiFix (http://mirifix.com). MiRiFix integrates predictions from Diana microT 3.0 [18], Target Scan 5.1 [19], microRNA.org (2008 release; [20]) and PicTar [21], as well as RegRNA [22], Rna22 [23], FindTar3 (http://bio.sz.tsinghua.edu.cn/) and MiRTif, which is a support vector machine-based miRNA-target filtering-system to distinguish true predicted target sites from false ones [24]. miRNA expression profiling data were accessed through MirZ [25] and mimiRNA [26].

### Statistical Analysis

Chi-square tests were performed to compare the allelic distribution of SNPs between FRDA patients and controls. Student-t test was used to validate the statistical significance in luciferase reporter assays.

## Results

### Association between SNPs in the 3′-UTR of Frataxin and FRDA

**Figure 1:**
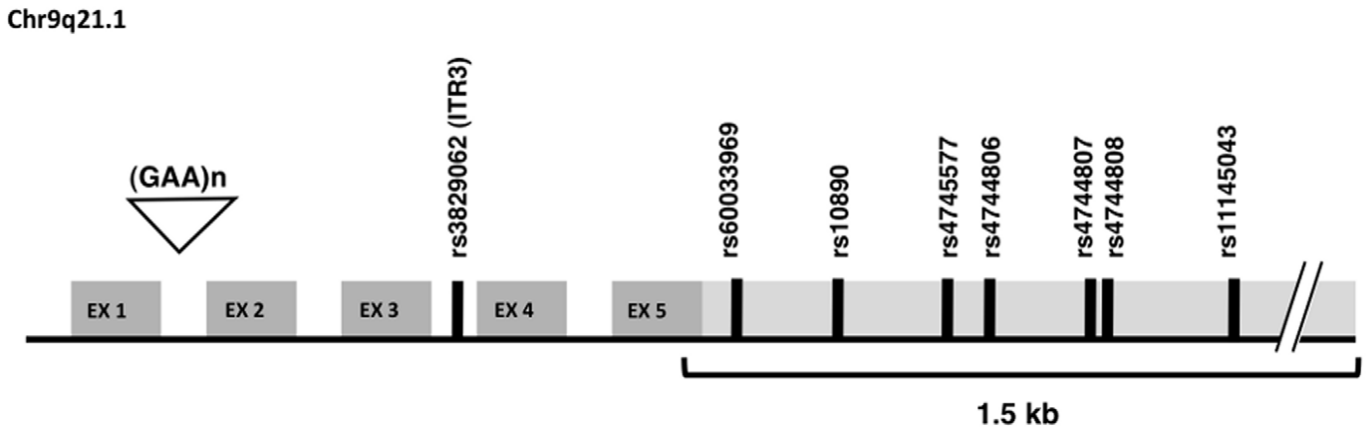
Schematized representation of the genomic structure of the *FXN* gene. The pathogenic expansion of the GAA repeat within intron 1 is indicated by a triangle, exons by grey boxes, respectively dark when translated and light when untranslated. Short genetic variations are indicated as black bars. The *FXN* 3′-UTR region, which was sequenced is highlighted by a square bracket.

To assess the existence of variants in the 3′-UTR of the *FXN* gene, we sequenced 1.5 kb of the gene 3′ terminus, which encompassed the 1451 bp of its 3′-UTR region, in a cohort of 57 FRDA patients and 58 controls (Figure 1). No mutations were found. Nineteen short genetic variations, which were already annotated in the dbSNP database were explored in our cohort in addition to rs3829062 (ITR3), a marker from the FRDA risk haplotype, already found to be associated to FRDA [5], [7], [27]. Of those 19, 12 variations were monomorph whilst seven variations, namely rs60033969, rs10890, rs4745577, rs4744806, rs4744807, rs4744808 and rs11145043 were biallelic markers as in the HapMap CEU population panel (Figure 1).

**Figure 2:**
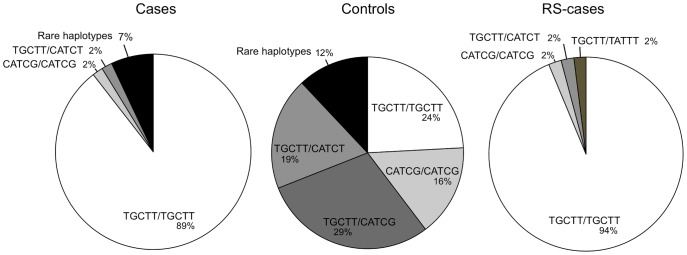
Distribution of the most common haplotypes. Genotype frequencies of haplotypes in cases, controls and the replication study cases (RS-cases) are plotted as pie charts for the most common haplotypes of the *FXN* 3′-UTR (TGCTT, CATCG, CATCT). Haplotype TATTT was uniquely found among RS-cases.

We calculated allelic frequencies of those polymorphic 3′-UTR variations in our cohorts of FRDA patients and controls (Table 1). Complete association was found between rs60033969 and rs10890, and between rs4744807 and rs4744808, respectively. Thus, results for rs60033969 and rs4744807 were not presented. All typed SNPs were significantly associated to FRDA with rs4744806 being even more strongly associated than ITR3 (Table 1). Patients were mostly homozygous for all SNPs with frequencies ranging from 93 to 98.2% among the different SNPs (Table 2). Conversely, homozygosity was under-represented among controls, ranging from 15.5 to 46.5% (Table 2). These SNPs from the 3′-UTR (rs10890, rs4745577, rs4744806, rs4744808 and rs11145043) defined several haplotypes (Table 3), which were differently distributed between cases and controls (Figure 2). We found that the vast majority of patients, i.e. 89%, were homozygous for the T-G-C-T-T haplotype versus 24% of control subjects (Figure 2). Of note, genotype frequencies of the haplotypes in our control cohort comply with Hardy-Weinberg equilibrium proportions, and were closest to the ones of Western European descent population available from 1000 Genomes project.

**Table 1:** Genetic association of ITR3 and SNPs of the *FXN* 3′-UTR with FRDA haplotype in cases versus controls.

	Frequency		?^2^	*P*-value
	Cases, n = 114 (%)	Controls, n = 116 (%)		
rs3829062 (ITR3)				
C	113 (99.1)	71 (61.2)	51.66	6.58×10^−13^
T	1 (0.9)	45 (38.8)		
rs10890				
C	4 (3.5)	53 (45.7)	54.878	1.28×10^−13^
T	110 (96.5)	63 (54.3)		
rs4745577				
G	109 (95.6)	63 (54.3)	52.013	5.51×10^−13^
A	5 (4.4)	53 (45.7)		
rs4744806				
C	111 (97.4)	63 (54.3)	57.871	2.80×10^−14^
T	3 (2.6)	53 (45.7)		
rs4744808				
T	108 (94.7)	60 (51.7)	54.024	1.98×10^−13^
C	6 (5.3)	56 (48.3)		
rs11145043				
G	2 (1.8)	40 (34.5)	41.259	1.33×10^−10^
T	112 (98.2)	76 (65.5)		

**Table 2:** Genotype frequencies of the five SNPs for the *FXN* 3′-UTR in cases, cases from the replication study (RS-cases), and controls.

	Frequency		
	Cases, n = 57 (%)	RS-cases, n = 47 (%)	Controls, n = 58 (%)
rs10890			
CC	1 (1.8%)	1 (2.1%)	11 (19%)
TT	54 (94.7%)	45 (95.8%)	16 (27.6%)
CT	2 (3.5%)	1 (2.1%)	31 (53.4%)
rs4745577			
GG	53 (93%)	44 (93.6%)	16 (27.6%)
AA	1 (1.8%)	1 (2.1%)	11 (19%)
GA	3 (5.2%)	2 (4.3%)	31 (53.4%)
rs4744806			
CC	55 (96.4%)	44 (93.6%)	16 (27.6%)
TT	1 (1.8%)	1 (2.1%)	11 (19%)
CT	1 (1.8%)	2 (4.3%)	31 (53.4%)
rs4744808			
TT	53 (93%)	45 (95.8%)	15 (25.9%)
CC	2 (3.5%)	1 (2.1%)	13 (22.4%)
TC	2 (3.5%)	1 (2.1%)	30 (51.7%)
rs11145043			
GG	1 (1.8%)	1 (2.1%)	9 (15.5%)
TT	56 (98.2%)	46 (97.9%)	27 (46.5%)
GT	0 (0%)	0 (0%)	22 (38%)

**Table 3:** Frequencies of the haplotypes comprising the five SNPs of the *FXN* 3′-UTR.

Haplotype	Frequency	
rs10890- rs4745577-rs4744806-rs4744808-rs11145043	Cases, n = 114, (%)	Controls n = 116, (%)
T-G-C-T-T	106 (93)	59 (50.9)
C-A-T-C-G	2 (1.8)	37 (31.9)
C-A-T-C-T	1 (0.9)	15 (12.9)
T-G-C-C-T	2 (1.8)	1 (0.9)
C-G-C-C-T	1 (0.9)	0 (0)
T-A-C-T-T	2 (1.8)	0 (0)
T-G-C-C-G	0 (0)	3 (2.6)
C-A-T-T-T	0 (0)	1 (0.9)

Forty-seven adult patients from Reunionese island, referred to as the replication study cases (RS-cases), were similarly genotyped (Table 2). 94% of this population was found to be homozygous for the T-G-C-T-T haplotype (Figure 2). This result further emphasizes our finding that the 3′-UTR haplotype, which we termed FRDA-3′-UTR for simplicity, expands the FRDA risk haplotype to the 3′-UTR.

### Functionality Assessment of the FRDA Haplotype and miRNA Targeting Predictions

**Figure 3:**
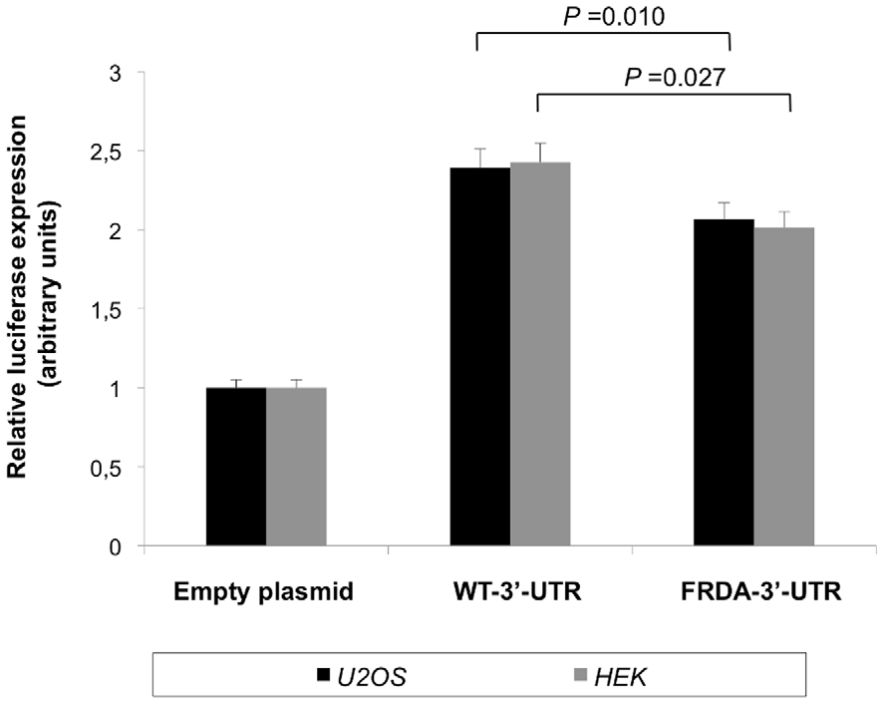
Functional assessment of FRDA-3′-UTR versus WT-3′-UTR. U2OS (black bars) and HEK-293 (grey bars) cells were transfected with luciferase reporter gene system, respectively 150 ng of empty plasmid or plasmid WT-3′-UTR or plasmid FRDA-3′-UTR. Histograms show the *Renilla* luciferase activity (normalized to firefly luciferase and to the mock transfected cells) following transfection of each plasmid into both cell lines. All results represent mean ± SEM of three independent experiments, each in triplicate. *P<0.05, Student-t test.

**Figure 4:**
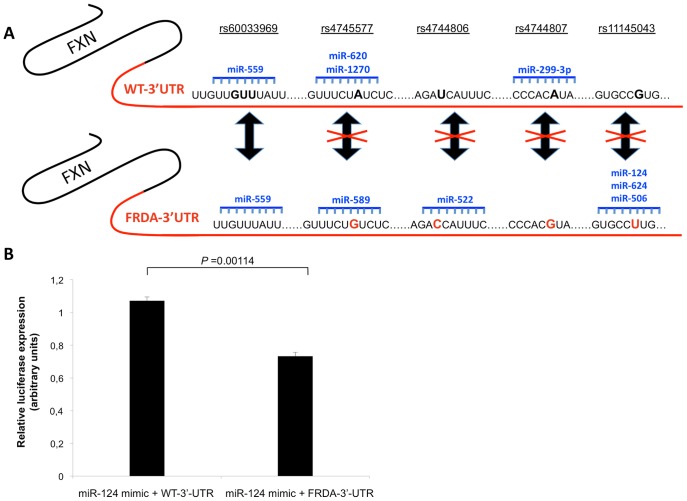
miRNAs targeting of FRDA-3′-UTR. A) Schematic representation of the nine miRNAs predicted to target *FXN* 3′-UTR dependengly on the haplotype: WT-3′-UTR (upper panel) or FRDA-3′-UTR (lower panel). The SNPs genotyped in the frataxin 3′-UTR are indicated in bold letters. Double arrows indicate whether the SNP is targeted by the same miRNA. Crossed double arrows indicates that the SNP is targeted by different miRNAs dependingly on the allele. B) Histograms illustrate the *Renilla* luciferase activity (normalized to firefly luciferase) following co-transfection of 10 nM miR-124 mimic with 150 ng of the indicated reporters into HEK 293 cells (*n* = 3). All results represent mean ± SEM of three independent experiments, each in triplicate. *P<0.05, Student-t test.

To investigate what type of regulation confers the FRDA-3′-UTR haplotype to the frataxin protein level, we transfected the FRDA-3′-UTR as compared to the alternative 3′-UTR-haplotype configuration, i.e. C-A-T-C-G, referred to as the WT-3′-UTR. We repeatedly found in U2OS as in HEK293 cells that the FRDA-3′-UTR affected the levels of frataxin through a significant decrease of 1.2-fold (Figure 3, for U2OS *P*-value = 0.001; for HEK293 *P*-value = 0.027; n = 3). Thus, the *FXN* 3′-UTR harboured at the homozygous state by the vast majority of patients may contribute to lower levels of frataxin in addition to the effect of the expanded mutation. Subsequently, we reasoned that the difference observed in the regulatory potency of FRDA haplotype versus control haplotype might at least partially be due to a distinct miRNA targeting. In order to test the hypothetical involvement of miRNAs in the regulation of frataxin, we first screened miRNA target sites of the *FXN* 3′-UTR. To this purpose, we used our in-house developed bioinformatic tool MiRiFix, and found prediction for 19 miRNAs at 15 target sites. Five of those 15 target sites overlapped one of the genotyped variations (Table 4). Based on our predictions, nine miRNAs differentially targeted the *FXN* gene, dependingly on the allele: hsa-miR-559, hsa-miR-589, hsa-miR-1270, hsa-miR-620, hsa-miR-522, hsa-miR-299-3p, hsa-miR-506, hsa-miR-124 and hsa-miR-624 (Table 4, Figure 4A). In particular, we found that variations rs4744806 and rs11145043 created novel target sites for hsa-miR-522, hsa-miR-506, hsa-miR-624 and hsa-miR-124 in the FRDA-3′-UTR, which were not predicted with WT-3′-UTR (Figure 4A). Finally, using training sets of experimentally validated predictions, each of those miRNA:*FXN* duplexes was confirmed but hsa-miR-1270 (Table 4). Our results suggest that miRNA targeting of *FXN* 3′-UTR is differentially affected by the different haplotypes.

**Table 4:** Computational analysis of miRNA targeting on *FXN* 3′-UTR.

SNP	Targeted allele	PredictedmiRNA	MirTif	
			Result	Score SVM
rs60033969	deletion	hsa-miR-559	True	1.6667198
	GTT	hsa-miR-559	True	1.6667198
rs4745577	G	hsa-miR-589	True	0.056741344
	A	hsa-miR-1270	False	-0.025866646
	A	hsa-miR-620	True	1.4955749
rs4744806	C	hsa-miR-522	True	0.086234009
	T	–	–	–
rs4744807	G	–	–	–
	A	hsa-miR-299-3p	True	0.11721095
rs11145043	T	hsa-miR-506	True	1.4271538
	T	hsa-miR-124-3p	True	1.2504418
	T	hsa-miR-624	True	3.1861664
	G	–	–	–

### 3′ -UTR of Frataxin as a Target of miRNA Regulation

We searched for the miRNA(s) whose pattern of expression showed more consistency with the phenotype. To this end, we searched for the miRNAs that were endogenous to either cell lines used for functional assessment of the 3′-UTR. Intersecting both patterns from public databases revealed miR-124 as the only common miRNA. We thus chose to assess the regulation by hsa-miR-124. Interestingly, miR-124 is also known as the most abundant miRNA in the nervous system making it worth studying in FRDA (reviewed by [28]). Overexpression of hsa-miR-124 in HEK-293 cells led to a reproducible and significant 32%-decrease of luciferase activity in cells transfected with plasmid FRDA-3′-UTR compared to cells transfected with plasmid WT-3′-UTR (Figure 4B, *P*-value = 0.00114; n = 3). These data are consistent with the computational findings of a specific targeting of miR-124 at the level of FRDA-3′-UTR, suggesting a post-transcriptional regulation of frataxin mediated by differential miRNA targeting.

## Discussion

Accumulating evidence suggests that variations at 3′-UTR may associate with the susceptibility to several diseases, including cancer, infectious diseases and neurological disorders [29]–[32]. In the *FXN* gene, the size of GAA expansion does not fully relate to the frataxin levels whether in patients and in healthy individuals [6], [9], [33]. In this context, we provide the first systematic analysis of the *FXN* 3′-UTR in patients with Friedreich ataxia and its regulation on frataxin levels. Sequencing analysis of the *FXN* 3′-UTR in a cohort of 57 children diagnosed with FRDA, confirmed in a cohort of 47 adult patients, allowed identification of a haplotype comprising seven informative short polymorphic variations. We demonstrated that this FRDA-3′-UTR haplotype further completes the founder haplotype for the GAA repeat expansion in Friedreich ataxia, and may thus represents a new molecular marker for FRDA. Importantly, we further established that this haplotype harboured by FRDA patients was associated with specific post-transcriptional regulation that likely influences frataxin level.

Numerous studies have shown the influence of SNPs on miRNA targeting [14]. It is interesting to note that miRNAs have been involved in neurodegenerative diseases caused by triplet expansions. Under this category fall different types of spinocerebellar ataxias (SCA), which are caused by a CAG repeat expansion within the coding region of the diseased gene, resulting in a polyglutamine domain that confers a toxic gain-of-function onto the protein. For instance, in SCA-3, deficiency of Dicer, a key enzyme in miRNA maturation, was associated with increased toxicity of Ataxin-3, the protein responsible for the neurodegeneration [34]. Moreover, miR-19, miR-101, miR-130 and lately miR-144 were found to co-regulate ATXN1, which is involved in spinocerebellar ataxia type 1 (SCA-1) [35], [36]. Here, we provide the evidence of a miRNA-based regulation of FRDA-causal gene, frataxin, but also unravel the specificity of regulation through the 3′-UTR that is achieved by the FRDA-3′-UTR haplotype, highly associated to FRDA. Future work is needed to study this miRNA-based regulation of frataxin in a system, which would be related to the FRDA context. Noteworthy, in either cohorts of patients, the few individuals that were not homozygous for the FRDA-3′-UTR presented an unusually mild clinical outcome (data not shown). We speculate that genotype/phenotype correlation at the level of *FXN* 3′-UTR notably occurs through a different combinatory set of miRNAs that could influence the phenotype spatially and temporally wise.

Our study further introduces the idea that miR-124 specifically regulates *in vitro* the FRDA-3′-UTR. miR-124 is among the miRNAs the most enriched in the central nervous system, where it plays a crucial role in neurogenesis and neuronal function (reviewed by [28]). Recently, miR-124 was found as overexpressed in FRDA patient cells [17]. As such, the specific regulation of FRDA-3′-UTR by miR-124 likely plays a role in the neuropathology of Friedreich ataxia. Sensitivity of subsets of neurons to the deficit of frataxin may indeed depend on the effectiveness of miR-124 targeting. To this purpose, induced pluripotent stem cells-derived neurons, which are derived from FRDA patients represent an interesting tool for assessing the functional consequences of modulating miR-124 in a disease context [37], [38]. In the future, the involvement of miRNAs in a FRDA-specific regulation of frataxin may provide a rationale for miRNA-based therapies in Friedreich ataxia. Yet much effort is required to characterize in greater depth the regulation of frataxin in the disease tissues of FRDA patients.
